# Unlocking the Therapeutic Potential of Irisin: Harnessing Its Function in Degenerative Disorders and Tissue Regeneration

**DOI:** 10.3390/ijms24076551

**Published:** 2023-03-31

**Authors:** Yuwei Zhang, Lizhen Wang, Hongyan Kang, Chia-Ying Lin, Yubo Fan

**Affiliations:** 1 Key Laboratory for Biomechanics and Mechanobiology of Ministry of Education, Beijing Advanced Innovation Centre for Biomedical Engineering, School of Biological Science and Medical Engineering, Beihang University, Beijing 100083, China; 2 Department of Biomedical, Chemical & Environmental Engineering, University of Cincinnati, Cincinnati, OH 45267, USA; 3 Department of Orthopaedic Surgery, University of Cincinnati, Cincinnati, OH 45267, USA; 4 Department of Neurosurgery, University of Cincinnati, Cincinnati, OH 45267, USA; 5 School of Engineering Medicine, Beihang University, Beijing 100083, China

**Keywords:** FNDC5, irisin, degenerative disease, regeneration, tissue repair

## Abstract

Physical activity is well-established as an important protective factor against degenerative conditions and a promoter of tissue growth and renewal. The discovery of Fibronectin domain-containing protein 5 (FNDC5) as the precursor of Irisin in 2012 sparked significant interest in its potential as a diagnostic biomarker and a therapeutic agent for various diseases. Clinical studies have examined the correlation between plasma Irisin levels and pathological conditions using a range of assays, but the lack of reliable measurements for endogenous Irisin has led to uncertainty about its prognostic/diagnostic potential as an exercise surrogate. Animal and tissue-engineering models have shown the protective effects of Irisin treatment in reversing functional impairment and potentially permanent damage, but dosage ambiguities remain unresolved. This review provides a comprehensive examination of the clinical and basic studies of Irisin in the context of degenerative conditions and explores its potential as a therapeutic approach in the physiological processes involved in tissue repair/regeneration.

## 1. Introduction

Physical activity is a crucial aspect of human life, as suggested by the evolutionary theory linking basal physical activities to survival [1,2]. Compelling evidence from recent decades shows that exercise protects against degenerative conditions such as muscle atrophy [3], osteoporosis [4], Alzheimer’s disease (AD), and Parkinson’s disease (PD) [5], as well as slow their progression in individuals who have already been diagnosed. Exercise has also been linked to improved tissue growth and renewal in the regenerative aspect, such as increased myogenesis [6] and osteogenesis [7], as well as better neurogenesis [8], which may further contribute to overall health and a mitigated risk of degenerative disease. Molecular effectors involved in exercise-related benefits have been identified through advanced molecular techniques.

In 2012, Fibronectin domain-containing protein 5 (FNDC5), an underappreciated transmembrane protein, was discovered as the precursor of Irisin, a myokine primarily expressed in skeletal muscle during exercise [9]. It promotes the browning of white adipose tissue and activates thermogenesis in response to mechanical stimuli like exercise, following the upregulation of peroxisome proliferator-activated receptor gamma coactivator-1-alpha (PGC-1α) [9]. Since the groundbreaking discovery of Irisin, scientists have been captivated by this “golden rush”, as evidenced by a steady annual increase in the number of publications on this topic, with over thousands of studies published to date. Primarily, Irisin has garnered significant research attention for its role in regulating energy metabolism and metabolic disorders such as obesity and diabetes mellitus, which are closely linked to physical activity levels [10,11]. The ability of Irisin to induce positive effects of exercise at the molecular level has prompted further investigation into its pathobiological roles, clinical significance, and therapeutic potential in various diseases, encompassing not only metabolic disorders but also degenerative conditions [12].

**Figure 1:**
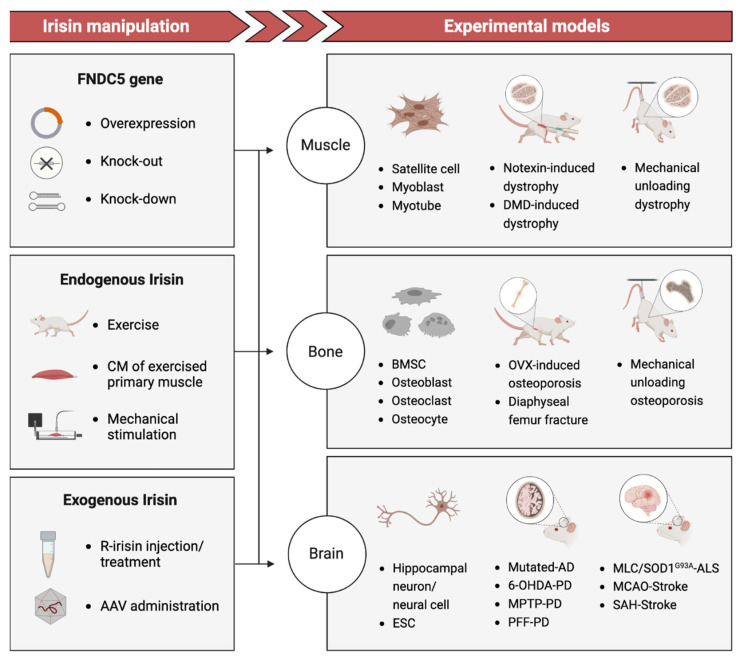
Basic studies: Irisin manipulation via gene regulation/exercise/mechanical stimulation/recombinant in experimental models including tissue engineering and animal models. FNDC5: Fibronectin domain-containing protein 5; Gene overexpression includes retinoic acid (RA)-induced FNDC5 overexpression [17] and muscle creatine kinase (MCK) promoter-induced overexpression [18]. CM: Culture media. R-Irisin: Recombinant Irisin from a commercial agent or produced in HEK 293 cells using DNA plasmid. AAV: Adeno-associated virus. Notexin-induce dystrophy: An experimental study of muscular injury repair caused by toxicity [16]. DMD-induced dystrophy: A popular model for studying Duchenne muscular dystrophy (DMD) mutation caused muscle atrophy [19]. BMSC: Bone marrow stromal cell. OVX-induced osteoporosis: A model to bone resorption/bone loss via null of ovariectomy (OVX) [20]. Diaphyseal femur fracture: a surgery performed to induce bone fracture and thus study bone repair [15]. Mutated-AD: An Alzheimer’s disease (AD) model of transgenic mice (APP/PS1M146L) [21]. 6-OHDA-PD: A Parkinson’s disease (PD) model induced by 6-hydroxydopamine [22]. MPTP-PD: A PD model induced by 1-methyl-4-phenyl-1,2,3,6-tetrahydropyridine (MPTP) [23,24]. PFF-PD: α-syn preformed fibril (PFF) mouse model of sporadic PD [25]. MLC/SOD1G93A-ALS: An amyotrophic lateral sclerosis (ALS) transgenic mice model (MLC/SOD1G93A) carrying a mutated superoxide dismutase 1 (SOD1), avoiding motor-neuron involvement [26]. MCAO-Stroke: A middle cerebral artery occlusion (MCAO) model used to produce cerebral ischemia after stroke in mice [27,28]. SAH-Stroke: A stroke model as Subarachnoid hemorrhage (SAH) is a devastating form of stroke [29].

Clinical studies have explored the correlation between plasma Irisin levels and degenerative disorders, utilizing a range of assays such as antibody-based methods (Western Blot, ELISA, Protein Liquid Chip Assay) and label-free methods (Quantitative Mass Spectrometry) to detect Irisin concentration [13]. While these investigations have directly provided initial evidence of how the serum level of Irisin is associated with the gradual deterioration of tissues and organs over time, the lack of reliable measurements for endogenous Irisin has resulted in numerous contradictions and uncertainties. However, Basic studies (Figure 1) using disease-mimicking animal models and tissue-engineering models to manipulate Irisin levels via recombinant Irisin (r-Irisin) administration or gene gain/loss of function have demonstrated the regenerative potential of Irisin treatment in reversing functional impairment and potentially permanent damage [14,15,16]. The underlying mechanism by which Irisin promotes tissue regeneration appears to exhibit variability on a case-by-case basis.

This article aims to comprehensively and systematically review the clinical and basic studies of Irisin in the context of degenerative conditions. Furthermore, we explore and discuss the physiological processes involved in deteriorated or damaged tissue, wherein Irisin is hypothesized to act as a regenerative effector and facilitates tissue regeneration.

## 2. Molecular Effectors and Mechanisms Involved in Exercise-Related Benefits

During exercise, the body undergoes significant structural and functional changes that are responsible for the numerous beneficial effects of physical activity. These adaptations are primarily driven by various types of mechanical signals, such as stretching, compression, shear stress, and fluid flow-induced stress [30]. The conversion of these mechanical stimuli into biochemical signals and further cellular responses is known as mechanotransduction, a critical process that mediates tissue homeostasis and repair [31]. Here, we explore the transduction of mechanical signals, particularly in the musculoskeletal system, and the subsequent activation of sequential signaling cascades, exercise mimics in diverse tissues to regulate disease prevention and tissue regeneration.

### 2.1. Mechanical Signals Transduction

In nature, the musculoskeletal system plays a crucial role in facilitating movement and safeguarding vital organs. Consequently, the cells within the muscles and bones serve as the primary sites for detecting mechanical stimuli through various sensors such as ion channels, integrins, and other transmembrane complexes, which predominantly convert physical distortions into electrical or biochemical signals.

#### 2.1.1. Mechanical Signals to Cellular Signals Transduction

As a major type of mechanosensors, ion channels primarily adapt their distinctive structures in response to mechanical forces. Upon gating (open and close), they allow the nonselective flux of ions such as cations into the cell, transducing mechanical stimuli into electrochemical signals. Piezo ion channels have emerged as pivotal molecular detectors of mechanical forces, drawing increasing attention for their regulatory roles in osteocyte [32,33], bone marrow mesenchymal stem cell (BM-MSC) [34], osteoblast and osteoclast [35,36] function in bone. Among these, the expression of Piezo1 in osteocytes and its critical involvement in mechanotransduction have been emphasized by numerous studies. Notably, Li et al. revealed that Piezo1 expression is upregulated in Mlo-y4 osteocytes under fluid shear force in in vitro research, and its deletion in osteocytes and osteoblasts results in reduced bone mass via Wnt signaling [32]. Furthermore, the activation of Piezo1 in osteocytes by mechanical stretch has been shown to mediate protein kinase B (Pkb or Akt) signaling phosphorylation [33]. According to an in vitro investigation, Piezo1 can be induced by mechanical stimulation of hydrostatic pressure to enhance the differentiation of BM-MSCs into osteoblasts through bone morphogenetic protein 2 (BMP2) upregulation [34]. In osteoblasts, Piezo1 serves as a crucial mechanical sensor to transduce fluid shear stress [37,38], extracellular matrix stiffness [38], and gravity [35,36], which can activate the Piezo1- Yes-associated transcriptional regulator 1 (YAP1)-collagen pathway or cooperate with Piezo2 to activate the nuclear factor of activated T-cells (NFATc)/YAP1/β-catenin transcription factor complex, contributing to osteogenic formation. However, the role of Piezo ion channels in skeletal muscles is not well-understood yet and there are disagreements in the existing literature [39,40].

Focal adhesion (FA) is another significant type of mechanosensors that transfers mechanical stimuli from the extracellular environment to the cellular cytoskeleton. Dynamically built and formed by transmembrane proteins named integrins, FAs are composed of extracellular domains that bind to ligands and intracellular domains that interact with the cytoskeleton and signaling molecules, regulating cellular functions. In the realm of load-induced bone formation, the integrin-based adhesion complex holds significant importance and is ubiquitously expressed across all types of bone cells [41,42]. However, since integrins lack inherent enzymatic activity, the transmission of signals through focal adhesion hinges upon associated molecules that trigger downstream signaling events such as intracellular calcium release, tyrosine kinase and MAP kinase activation [43,44]. The Focal adhesion kinase (FAK) family, comprising of FAK and proline-rich tyrosine kinase 2 (PYK2), represents significant tyrosine kinases activated upon integrin engagement, responsible for the regulation of osteoclast adhesion and bone resorption [45]. Additionally, this family plays a pivotal role in Insulin-like growth factor 1 (IGF-1)-induced muscle hypertrophy signaling through a tuberous sclerosis complex 2 (TSC2)/mammalian target of rapamycin (mTOR)/ribosomal S6 kinase 1 (S6K1) dependent signaling pathway [46].

#### 2.1.2. Cell-Cell Mechanotransduction

Apart from mechanosensors such as ion channels and integrins that convert external mechanical signals into intracellular electrical or biochemical signals, certain transmembrane structures act as cell-cell communicators. These structures anchor onto the surface of another cell and act as receptors, receiving signals in the form of ligands from sensing cells. Once received, these signals are passed into host cells to modulate cell growth and renewal. Notable examples of such transmembrane structures include connexins [47], pannexins [48], notch [49,50], and low-density lipoprotein receptor-related proteins 5/6 (Lrp5/6) [51,52,53].

### 2.2. The Activation of Sequential Signaling Cascades

Mechanosensors enable cells to activate sequential signaling cascades and express downstream target genes in response to mechanical stimulation. These responses exhibit commonalities, including in cell types such as satellite cells, myoblasts, osteocytes, osteoblasts, osteoclasts, and neurons, and are typically mediated by several key transmitters involved in different canonical signaling pathways.

Emerging evidence suggests that Wnt/Lrp5 signaling is susceptible to mechanical loading, particularly in bones. Exercise-induced mechanical loading can downregulate the expression of negative regulators of the Wnt pathway, namely sclerostin (SOST) [32,54,55] and Dickkopf-related protein 1 (Dkk1) [56], in osteocytes. This downregulation, in turn, triggers the formation of new bone and prevents disuse osteoporosis, thus highlighting the profound impact of physical activity on skeletal health. The receptor for Wnt ligands, LRP5/6, plays a crucial role in transmitting Wnt/β-catenin signaling, which modulates the proliferation and activity of osteoblasts, and hence bone mass [57]. The mechanical significance of LRP5 has been demonstrated in LRP5^−/−^ mice, which exhibited almost complete inhibition of ulnar loading-induced bone formation compared to wild-type controls [58,59]. Recently, Zhong et al. reported that in vitro mechanical tension on osteoblasts upregulated LRP5 gene expression at 1, 3, and 5 h of loading [60].

The Phosphoinositide 3-kinase (PI3K)/Akt signaling pathway is another common mechanism that is predominantly regulated by mechanical stimuli in various tissues frequently seen in muscle [61,62] and brain [63,64]. Mechanical stress activates β1 Integrin, which in turn triggers integrin-linked kinases (ILK) [65] and focal adhesion kinase (FAK) [46,66], leading to the activation of the PI3K/Akt/mammalian target of rapamycin complex 1 (mTORC1) process. Furthermore, exercise is known to induce the growth factor-dependent PI3K/Akt/mTORC1 signaling axis to regulate tissue regeneration. It has been proposed that resistance exercise promotes muscle hypertrophy by increasing systemic growth factors such as IGF-1, which activate the PI3K/Akt/mTORC1 signaling axis to enhance muscle protein synthesis [46,67]. The exercise-induced growth factor BDNF also plays a crucial role in neuronal survival [68], promoting regeneration in injured brains [69] and resistance to degenerative disorders such as Alzheimer’s disease [63] via the PI3K/Akt signaling axis [68,70,71,72].

Upon the activation of mechanosensitive ion channels and subsequent recruitment of scaffolding proteins, members of the mitogen-activated protein kinase (MAPK) family, including extracellular signal-regulated kinases 1 and 2 (ERK1/2) [73], p38 MAPK [74], and c-Jun N-terminal kinase (JNK) [75], also participate in the process of mechanotransduction. Similar to PI3K/Akt signaling, exercise-induced various growth factors such as neuregulin 1 (NRG1) [76], transforming growth factor beta (TGF-β), and BMP [77] can trigger MAPKs signaling, which plays a vital role in exercise-induced myogenesis, osteogenesis and neurogenesis [73,78].

### 2.3. Exercise Mimics

The pursuit of orally active agents that mimic or potentiate the genetic effects of exercise has been a longstanding objective in the medical community, given the multiple benefits of exercise for general health. While this goal has proven elusive, natural extracts like resveratrol have been shown to enhance endurance [79]. As a key regulator of the adaptive response to exercise, PGC-1α plays an essential role in mediating the communication between exercise-induced muscle regeneration and mitochondrial biogenesis [80,81]. The aerobic benefits of resveratrol are thought to depend on the activation of the adenosine monophosphate-activated protein kinase (AMPK)/sirtuin 1 (SIRT1)/PGC-1α pathway in skeletal muscle [79]. Peroxisome proliferator-activated receptors (PPARs) are also known to interact with PGC-1α as part of exercise-induced physiological responses [82]. Consequently, AMPK and PPARs agonists have been proposed as promising exercise mimetics [83]. However, ongoing research into the regulatory mechanisms of these biomolecules has revealed other biosynthetic agents, such as adipokine (Adiponectin), cytokine (interleukin-6), and myokine (Irisin), which can also mimic the effects of exercise [9,11,84]. Although the potential exercise-induced protective and regenerative effects of biosynthetic agents remain subject to investigation, the emergence of Irisin as a novel target has garnered significant attention due to its apparent involvement in an array of diseases and metabolic conditions. The prevalent hypothesis and subsequent research suggest that Irisin, produced downstream of PGC-1α, is released from muscle in response to exercise and promotes browning and thermogenic response through uncoupling protein 1 (UPC1) upregulation in white adipose tissue (WAT) [9]. This mechanism has gained significant attention for its perceived ability to regulate disorders such as obesity and diabetes [85]. Meanwhile, numerous studies have highlighted Irisin’s potential as a preventative, interventive or treatment option for degenerative diseases [5,13,86,87].

## 3. Current Evidence: Irisin as a Potential Link to Degenerative Disorders

Degenerative diseases are a group of conditions that involve the gradual deterioration of tissues and organs over time, resulting in functional impairment and potentially permanent damage. These conditions typically become more prevalent as individuals age and can affect various parts of the body, such as muscles, bones, the brain, and the nervous system. Examples of typical degenerative conditions include muscle atrophy, osteoporosis, amyotrophic lateral sclerosis (ALS), AD, and PD. Currently, there is no cure for most degenerative diseases, and treatment options typically focus on managing symptoms and improving quality of life [88,89,90]. Considering the responsiveness of Irisin to exercise, researchers are highly motivated to explore the potential of this versatile myokine as a biomarker or preferred treatment option [91].

### 3.1. Muscle Atrophy

Skeletal muscle atrophy denotes the loss of muscle mass resulting from an imbalance between protein synthesis and degradation, leading to a reduced cross-sectional area of myofibers and diminished muscle strength [92]. Potential triggers of muscle wasting include long-term immobilization, malnutrition, severe injuries, and aging, as well as various serious and often chronic diseases, leading to increased morbidity, mortality, and decreased quality of life [93]. Thus, addressing muscle wasting and fatigue has become a significant clinical concern. Irisin has emerged as a promising biomarker for predicting the onset of muscle atrophy [16,94]. However, to develop this molecule as an effective therapeutic intervention, a comprehensive understanding of both the underlying molecular processes and clinical investigations is required.

#### 3.1.1. Clinical Studies

Back in 2014, An initial cohort investigation suggested that the levels of circulating Irisin are correlated with the gains in muscle mass and strength resulting from exercise training [10]. However, the limited sample size and outbalanced training regimens have weakened the convinces of this correlation between exercise-adaptive Irisin and enhancing muscle mass. In another study involving human subjects, a positive correlation was also observed between Irisin levels and grip strength (R = 0.526, *p* = 0.002) as well as leg strength (R = 0.414, *p* = 0.003) in the resistance training exercise group [95]. This finding suggests that Irisin levels may be indicative of muscle status, although the indirect nature of this evidence necessitates further investigation. A cross-sectional analysis of community-dwelling Koreans has provided compelling evidence supporting the potential utility of circulating Irisin levels as a novel biomarker for age-related muscle degeneration [86]. Notably, this study represents the first investigation of Irisin in a pathological context and directly implicates Irisin as a potential biomarker of muscle dysfunction with important implications for predicting the onset of sarcopenia and offering new avenues for monitoring age-related changes in muscle function. Parallel findings were reported in postmenopausal women with sarcopenia [96]. Nonetheless, the use of Irisin-based early screening as a staging tool for sarcopenia remains controversial in light of varying cohorts of older adults [97], obesity groups [98] or dialysis patients [99].

In order to fully elucidate the potential of Irisin as a biomarker and therapeutic target, further studies are needed to address the ambiguities surrounding the sarcopenia syndrome. This will require the standardization of parameters and techniques to more precisely define study cohorts, as well as the expansion of participant numbers and types.

#### 3.1.2. Basic Studies

A complex interplay of pathophysiological factors orchestrates molecular mechanisms leading to muscle-specific protein degradation [92]. Among these factors, maladapted anabolic proteins, such as myogenin [100], and IGF-1 [101], and catabolic proteins, including myostatin [102], act in concert to disrupt the delicate equilibrium between muscle synthesis and breakdown. Circulating Irisin levels have been reported as positively correlated with IGF-1 concentrations in humans [103]. Similarly, increased FNDC5 mRNA and Irisin secretion have been observed during myogenic differentiation in human myocytes, with upregulated PGC1α and myogenin expression [104]. In a mouse model featuring myostatin-knockout, elevated Irisin levels were observed in conjunction with increased musculature [105]. These findings initially implied that Irisin may play an important role in the regulation of muscle growth.

Irisin showed a pleiotropic role in muscle growth, stimulating IGF-1 and inhibiting myostanin via the extracellular ERK pathway [104]. R-Irisin therapy prevents and ameliorates disuse-induced muscle atrophy in a hind-limb suspended mice model by increasing muscle mass [94]. Subsequent research has disposed of the underlying mechanisms through which murine Irisin protein injection can increase myogenic differentiation and myoblast fusion, leading to significant hypertrophy in mice. Specifically, these effects are mediated through the activation of interleukin-6 (IL-6) signaling [16]. In the same study, they also found the regenerative role of Irisin as it triggered the satellite cell activation and reduced protein degradation to resist injury-induced muscle loss. In addition, muscle atrophy is characterized by a shift in the fiber-type composition, for instance, the loss of contractile proteins can predominantly affect the type II fast fibers during aging [106]. Correspondingly, a recent investigation showed a transition of fiber types, as an adaptive response to mechanical stimulation, is positively associated with the secretion level of Irisin [107].

Taken together, these findings suggest that Irisin/FNDC5 may play a pleiotropic role in inducing an exercise phenotype in muscle mass and musculature, providing valuable insights into the potential mechanisms underlying the effects of Irisin on muscle physiology, as well as highlighting the potential for Irisin-based interventions aimed at enhancing muscle regeneration. With additional research on human skeletal muscle, Irisin may hold the promise to pave the way for new interventions aimed at enhancing skeletal muscle function and improving overall health.

### 3.2. Osteoporosis

Osteoporosis is a skeletal disorder characterized by compromised bone strength resulting from the deterioration of bone mass, bone mineral density (BMD), and bone microarchitecture, which predisposes a person to an increased risk of fractures and disability [108]. Osteoporosis is a prevalent degenerative disorder that primarily affects the elderly population [109]. However, it can also manifest in young individuals with metabolic disorders [110], neurodegenerative diseases [111], cancer [112], and astronauts during long-time space exploration, as a result of microgravity [113]. While pharmacological intervention such as bisphosphonates, is an important treatment option for osteoporosis, there is growing concern about their long-term safety [114]. As an alternative, exercise has emerged as a potent non-pharmacological intervention for both bone prevention and recovery [115]. With the ability to replicate the effects of exercise, Irisin has been extensively studied as a potential non-invasive treatment for bone tissue, with promising prospects for future development.

#### 3.2.1. Clinical Studies

An early epidemiological analysis reported that circulating Irisin levels are associated with previous osteoporotic fractures in postmenopausal women [116]. Although this conclusion has been further validated in another 2 groups of postmenopausal women with either osteoporosis [117] or hip fracture [118]. However, it is still unclear whether this association is independent or if it is due to confounding factors, such as lower muscle mass. Singhal et al. provided important insights into the role of Irisin in bone health, particularly in the context of physical activity, as the levels of Irisin in this study were compared between adolescent female athletes and non-athletes, with results demonstrating that Irisin levels serve as a determinant of areal and volumetric BMD, as well as bone strength estimates, in athletes but not in non-athletes [119]. An inverse correlation has been observed between Irisin levels and vertebral fragility fractures in overweight cohorts, while no significant correlation is found with BMD or lean mass [120]. A noteworthy study has highlighted the therapeutic potential of Irisin by revealing elevated levels of Irisin in the blood during the bone healing process in patients, as well as by detecting the presence of Irisin receptors in their bone cells [121].

Collectively, numerous clinical evidences have disclosed a positive link between circulating levels of Irisin and bone formation/repair. Nonetheless, it is acknowledged that the group sizes in these studies are relatively limited, meanwhile, the reported reference values of Irisin levels are quantitatively inconsistent, varying widely from pg/mL to μg/mL in serum. These variations may be attributed to factors such as age, gender, body mass, pathological conditions, and, most likely, differences in methodology and study design.

#### 3.2.2. Basic Studies

The bone possesses an exceptional endogenous regenerative capacity that allows for scarless healing and the restoration of its previous mechanical function, even in challenging conditions such as fracture, advanced age or metabolic and degenerative disorders [122]. The four most crucial cells involved in bone regeneration are osteogenic cells, osteoblasts, osteocytes, and osteoclasts. Together, they constitute the basic multicellular unit (BMU) responsible for the dynamic maintenance of bone formation and bone resorption [123]. Irisin has been proven to possess the ability to regulate these four types of cells, tipping the balance of catabolic and anabolic responses [18,20,94,124,125,126].

Irisin exerts a potent anabolic impact on osteogenesis in vitro and in vivo. Specifically, both r-Irisin and culture–media-induced Irisin (CM-Irisin) enhance osteoblast differentiation and mineralization in bone marrow stromal cells (BMSCs) [125,126] and MC3T3 cells [127] at a concentration of 100 ng/mL, with accumulated collagen type 1 alpha-1 (Col1α 1) and alkaline phosphatase (Alp) in vitro. In vivo study conducted on young healthy mice found that r-Irisin improved cortical bone mass and mechanical properties. In an osteoporotic model induced by mechanical unloading, treatment with r-Irisin prevented bone density reduction and reversed cortical and trabecular BMD loss, indicating its potential to treat osteoporosis via bone formation. However, in another osteoporotic model induced by ovariectomy (OVX), Kim et al. disclosed that ablation of the FNDC5 gene can prevent OVX-induced osteoporosis via inhibition of bone resorption, as osteoblast number or bone formation rate remains the same [20]. Further evidence in the same study pointed out 6 daily injections of 1 mg/kg of Irisin unleashed the expression of sclerostin [20], an inhibitor of bone formation, while this inhibitor was suppressed with 4 weekly doses of 100 μg/mL Irisin [94]. Whether Irisin exerts catabolic or anabolic effects on the skeleton likely depends on the dosage and administration regimen. It is hypothesized that intermittent pulses of Irisin at lower doses, as seen during exercise, may provide anabolic benefits on bone, while high doses could lead to bone catabolism. Interestingly, both dosing patterns can prevent osteocyte osteolysis in the context of osteoporotic mice, demonstrating the ability of Irisin in regulating bone modeling [20,128]. Additional studies validated the effects of Irisin on osteoclastogenesis in vitro at a concentration of 2–10 ng/mL and increased bone resorption in vivo in the Fndc5-overexpressed mice model [18].

Together, these findings suggest the intricate coordination mechanisms of Irisin in bone metabolism and highlight the role of the exercise-mimetic myokine Irisin in bone tissue as a multi-play of different types of bone cells. However, exploring the effects of Irisin on the BMU, optimizing the in vivo dosage of Irisin, and developing authentic bone injury model to directly examine the regenerative function of Irisin in bone healing need further exploration.

### 3.3. Neurodegenerative Disease and Injury

Neurodegenerative disorders are characterized by the progressive loss of specific groups of neurons, resulting in a deterioration in intellectual and cognitive abilities [129]. Examples of such diseases include but are not limited to AD, PD, Huntington’s disease, and ALS, which vary in their pathophysiology and can cause life-threatening impairments in memory, movement, speech, and breathing [130]. Neurodegenerative diseases have become an increasingly prevalent issue, and despite extensive attempts into their mechanisms and potential solutions, finding an effective cure remains challenging [131]. Numerous health research agencies, including the US National Institute on Aging, have affirmed the significant impact of appropriate physical exercise on enhancing cognition, across various age groups [132]. Scientific literature also reports the positive effects of consistent, prolonged physical activity and exercise interventions on cognitive function [133,134]. As few treatments are available to approach cognitive impairment, exercise-mimicking Irisin holds great promise as a non-pharmaceutical intervention for neurodegenerative disease or damage [135].

#### 3.3.1. Clinical Studies

A noteworthy investigation has revealed a decline in FNDC5/Irisin levels within the brain and cerebrospinal fluid (CSF) of AD patients, but not in plasma, suggesting a distinct decrease within the central nervous system (CNS) [21]. This study also identified a positive correlation between age and CSF Irisin in non-demented individuals, but not in MCI and AD patients, implying that the age-related increase in brain FNDC5/Irisin might be an endogenous mechanism to tackle the challenges confronted by the aging brain. Likewise, other studies have indicated that the cognitive benefits of circulating Irisin may be weakened by pathological conditions induced by AD in the brain. The association between plasma levels of Irisin and cognition is significant in participants without AD, but lost significance in those with AD [136]. In addition, individuals with obesity and low serum Irisin levels exhibit neurocognitive deficits during a visuospatial working memory task, indicating a potential influence of Irisin on cognitive function in this population [137]. However, this conclusion can be misleading due to the confounding effect of obesity.

Due to its anabolic role in skeletal muscle, FNDC5/Irisin is believed to be linked to the pathophysiological process of ALS [138,139]. ALS patients present about four times higher levels of circulating Irisin compared to healthy controls, and Irisin is significantly associated with disease severity, respiratory function decline, and free fat mass level in ALS patients, particularly those with a hyper-metabolic status [140]. In addition, Irisin serves as an independent prognostic marker that enhances current risk stratification for stroke patients [141] who undergo complex pathophysiological processes that lead to cerebral injury after stroke, including excitotoxicity, oxidative stress, inflammation, and apoptosis. The CSF Irisin concentration derived from stroke patients is lower than those derived from controls [142]. Furthermore, Irisin appears to be a promising therapeutic approach for PD, as evidence suggests that exercise increases serum Irisin levels in PD patients, which is positively linked to improved balance function (BBS scores) and reduced risk of falls and postural instability Irisin exhibits neuroprotection by preventing mitochondrial damage in PD patients.

#### 3.3.2. Basic Studies

While cellular senescence can be a helpful response to injury, it can worsen age-related brain dysfunction when tissue regeneration is depleted or saturated in older individuals. FNDC5/Irisin appears to be expressed robustly not only in skeletal muscle but also in various regions of brain tissue [9,143].

The roles of Irisin in improving cognitive functions in clinical AD have already been described in previous clinical studies. Elevated circulating Irisin by peripheral delivery could pass through the blood–brain barrier and result in the enrichment of central Irisin. Emerging experimental models have been conducted and established their role in ameliorating both the cognitive deficit and neuropathology in AD animals. Loss- and gain-of-function studies demonstrate Irisin’s protective role in restoring memory and cognitive function impairment [21,144]. This could be attributed to the modulation of β-cleavage of the amyloid precursor protein, resulting in reduced Aβ production, by FNDC5 binding with the first 1-16 amino acids of amyloid precursor protein [145]. In addition, the benefits of exercise on cognition or memory function can be ascribed to the activation of the PGC-1α/FNDC5/brain-derived neurotrophic factor (BDNF) signaling via the AMPK pathway [21,146,147,148]. In a rat model of PD induced by 6-hydroxydopamine (OHDA), levels of PGC-1α, FNDC5, and BDNF in the striatum and hippocampus were negatively affected, leading to increased neurodegeneration. However, this decline was prevented by a 16-week treadmill training prior to the onset of PD [22]. Upon another toxin 1-methyl-4-phenyl-1,2,3,6-tetrahydropyridine (MPTP) induced PD rat model, Zarbakhsh et al. revealed that Irisin as a neurotrophic factor protects against dopaminergic neurons in the injured brain, suggesting its potential not only in the prevention of PD but also the prevention of neural damage in the brain [23,24]. The translational promise of Irisin has been highlighted in a recent study, in which Irisin prevents the formation of pathologic α-synuclein (α-Syn) and protects neurons against α-Syn preformed fibril (PFF) induced neurotoxicity in rats. Besides, elevating blood Irisin levels in mice prevents neurodegeneration and physiological deficits induced by α-syn preformed fibrils injection [25]. Camerino et al. proposed that Irisin may restore muscle-nerve communication as a critical link between muscle and CNS in ALS and a likely pharmacological target in an ALS mouse model [26].

Cerebral Ischemic stroke, the most common form of stroke, accounts for 80% of stroke cases [149]. In response to hypoxia and ischemia following ischemic stroke, the overproduction of reactive oxygen species (ROS) has emerged as a pivotal contributor to oxidative stress. Failure of endogenous antioxidant mechanisms to efficiently eliminate ROS exacerbates oxidative stress [150]. However, Irisin has been shown to protect against neuronal damage caused by oxidative stress by inhibiting the ROS- NLR family pyrin domain containing 3 (NLRP3) inflammasome signaling pathway [151]. Moreover, administration of Irisin has also been found to lower levels of oxidative stress biomarkers such as nitrotyrosine (NO2-Tyr), superoxide anion and 4-hydroxynonenal (4-HNE), highlighting its antioxidant characteristics [28]. Importantly, excessive oxidative stress can trigger neuronal apoptosis; yet in a mouse model of experimental cerebral edema stroke, Irisin has been shown to reduce apoptosis and increase cerebral cortex levels of BDNF, thus leading to brain damage attenuation [27]. Irisin also contributes to the neuroprotective effects of physical exercise against cerebral ischemia by mitigating the release of the pro-inflammatory cytokine tumor necrosis factor (TNF)-α via the Akt and ERK1/2 signaling pathways [28]. As another destructive form of stroke, Subarachnoid hemorrhage is relieved through the administration of exogenous Irisin, which provides neuroprotective effects including maintaining mitochondrial morphology and promoting mitochondrial biogenesis [29] Thus, the exercise-induced hormone Irisin plays vital neuroprotective roles against stroke-related pathophysiology, including oxidative stress, inflammation, and apoptosis.

Current evidence shed light on more about the neuroprotective role of Irisin as a transmitter of exercise defending neurodegeneration and destroying brain function. Optimization of Irisin delivery as a biologic therapy, such as adeno-associated virus (AAV) administration, holds promise for the treatment of PD and other neurodegenerative disorders [25]. To better understand the potential therapeutic use of Irisin in addressing neurodegenerative diseases and relevant injuries more investigations including more experimental models are to be conducted.

## 4. Is Irisin an Oracle to Tissue Repair/Regeneration?

The regenerative potential of exercise has been a focus of research in recent decades, as many adult human organs have limited regenerative capacity. Enhancing tissue regeneration is a major challenge in regenerative medicine. As previously discussed, Irisin, an exercise-mimetic with therapeutic potential for various degenerative conditions, may offer a pharmaceutical alternative for individuals who are unable to exercise as well as provide a new approach to combat injury or senescence. The following section explores more particular roles of Irisin in tissue repair/regeneration.

### 4.1. Role of Irisin in Regulating Inflammatory Responses

**Figure 2:**
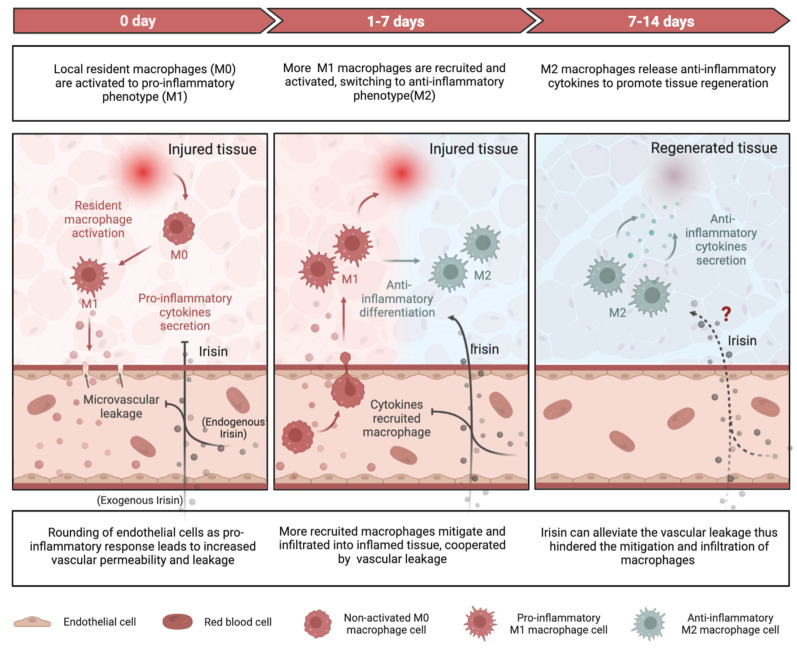
Schematic representing the anti-inflammatory function of Irisin during tissue repair. At the beginning of tissue repair, local resident macrophages (M0) are activated to pro-inflammatory phenotype (M1) macrophages, which release pro-inflammatory cytokines such as IL-6, IL-1β, and TNF-α, and change the endothelial to round-like shape, hence increase the vascular permeability and cause vascular leakage benefiting the mitigation and recruitment of more pro-inflammatory macrophages form peripheral blood into inflamed tissue. Irisin can blunt this acute-phase inflammatory response via downregulating pro-inflammatory cytokines and reducing vascular permeability. Furthermore, Irisin has been proven with the ability to stimulate anti-inflammatory macrophages (M2) polarization from M1, resulting in elevated anti-inflammatory cytokines secretion. However, it remains to be determined if Irisin directly mediates the M2 macrophages.

Inflammation plays a crucial role in both chronic and degenerative diseases, but it also facilitates regeneration in injured tissues by clearing damaged cells and promoting tissue regrowth [152,153]. Insufficient inflammation can lead to damage of tissues by harmful stimuli, while persistent unresolved inflammation can result in various pathologies, such as fibrosis [154] and cancer [155]. Recent research has elucidated the mechanisms by which Irisin, an exercise that resembles a mediator, alleviates inflammatory responses during tissue repair/regeneration at the molecular and cellular levels (Figure 2).

#### 4.1.1. Pro-Inflammation and Anti-Inflammation

After exercise, which is the primary trigger for Irisin production, the levels of pro-inflammatory cytokines such as TNF-α, interleukin-1 β (IL-1β), IL-6, and macrophage inflammatory protein 1α and 1β (MIP1α, MIP1α) decline in the bloodstream, while the levels of anti-inflammatory cytokines including interleukin-4 (IL-4), interleukin-10 (IL-10), interleukin-1RA (IL-1RA), and interleukin-13 (IL-13) rise [156,157,158,159]. Mazur et al. reported an anti-inflammatory action of Irisin downregulating IL-6 and TNF-α expression and secretion via inhibition of nuclear factor kappa B (NF-κB) in adipocytes [160]. Upon suppressing IL-1β, TNF-α, and IL-6 and toll-like receptor 4 (TLR4), which induces inflammatory response as sensing tissue injuries, Irisin can also mitigate lipopolysaccharide (LPS) induced liver or cardiac injury in vivo [161] and LPS-induced H9c2 cardiomyocyte injury in vitro [162]. Furthermore, coordinated with melatonin, Irisin could protect the heart against sepsis-induced myocardial depression via impeding the macrophage stimulating 1 (Mst1) and hence JNK pathway [163].

Irisin also possesses anti-inflammatory properties and remarkably alleviates neuroinflammation in brain injury with various causes. Irisin treatment significantly decreases the expression of IL-1β and TNF-α following ICH via the integrin αVβ5/AMPK signaling pathway [164]. Additionally, Irisin protects neurons by attenuating the secretion of pro-inflammatory cytokines such as TNF-α through Akt/ERK1/2 signaling [28]. Another pro-inflammatory oriented process, the activation of NLRP3 inflammasome when recognizing damaged proteins, is also inhibited by Irisin in ischemic conditions [151,165]. In particular, Irisin treatment significantly suppressed pro-inflammatory cytokine expression by deactivating the MAKP pathway in hypoxia/reoxygenation-induced neural injury [166].

#### 4.1.2. Macrophage Function

Irisin regulates macrophage function by mitigating the excessive production of ROS, indicating its potential anti-inflammatory properties [167]. Similarly, this anti-inflammatory function has been emphasized as Irisin blocks the production of pro-inflammatory cytokines including IL-1β, TNFα, IL-6, and MCP-1 in RAW 264.7 macrophages via mitogen-activated protein kinases (MAPK) pathway [168]. M1-type macrophages secrete pro-inflammatory cytokines such as TNF-α and IL-1β, whereas M2-type macrophages produce anti-inflammatory cytokines such as IL-10 [169]. In the context of obesity-induced chronic inflammation, the administration of exogenous FNDC5 was found to inhibit LPS-induced differentiation of M1-type macrophages, while the deficiency of FNDC5 promoted such differentiation [170,171]. The impact of Irisin on M1 macrophages has been clarified, but it remains to be determined if Irisin directly mediates the M2 macrophages.

#### 4.1.3. Vascular Permeability

Irisin has the capacity of enhancing vascular permeability via AMPK phosphorylation, as documented in several research publications [104,127]. Through AMPK signaling, both cell division cycle 42 (Cdc42) and Rac family small GTPase 1(Rac1) are activated, which in turn reinforce the endothelial barrier function and prevents microvascular leakage during inflammation [172]. The src family kinases (SFKs) mediate the vascular leakage through another mechanism-rounding of endothelial cells in response to various stimuli including LPS [173]. Irisin has been found to suppress the tyrosine kinase activity of SFKs, thereby curtailing the downstream increased vascular permeability when disposing of inflammatory responses [172]. By virtue of its interactions with either SFKs or AMPK signaling, Irisin can attenuate vascular permeability and impede the infiltration and recruitment of macrophages or leukocytes into inflamed tissues, culminating in a dampened inflammatory response [174].

Overall, Irisin plays a protective role in reducing severe inflammation by decreasing pro-inflammatory cytokines, increasing anti-inflammatory cytokines, promoting M2-type macrophage polarization, and inhibiting vascular permeability to prevent immune-cell infiltration into damaged tissues (Figure 2). The essentiality of Irisin’s anti-inflammatory role in repairing and regenerating adipose, cardiovascular, liver, and brain tissues is well-established. However, for other types of tissue damage, such as muscle injury, further research is required.

### 4.2. Role of Irisin in Coordinating Proliferation, Differentiation and Apoptosis

In various types of stem cells and precursor cells, FNDC5/Irisin has been shown to promote proliferation, differentiation, and maturation, facilitating myogenesis, osteogenesis, and neurogenesis in both physiological and pathological conditions. In this chapter, we particularly focus on the regenerative role of Irisin in the context of tissue self-renewal/repair with disease or damage.

#### 4.2.1. Myogenesis

Regeneration of adult skeletal muscle is an asynchronous process that involves the activation, proliferation, and fusion of satellite cells to form new muscle fibers [175]. Irisin has been found to play a role in this process by participating in myogenesis, including the activation of satellite cells, myogenic differentiation, and hypertrophic protein synthesis during the recovery or healing of atrophic muscle.

Satellite cells comprise a heterogeneous population of muscle stem cells that are typically activated by traumatic stimuli, exercise, or growth signals [176]. Following activation, these cells undergo either symmetric or asymmetric divisions, resulting in an increased pool size or committed satellite cell progenitors, respectively, which are responsible for myogenesis [177]. Subsequently, myogenic progenitors proliferate before differentiating, either by fusing with each other or with damaged fibers, leading to the restoration of fiber function and integrity, thereby protecting against degeneration or injury in adult muscle [178]. A dosage of 2.5 μg/g Irisin intraperitoneally is able to awaken quiescent satellite cells with upregulated MyoD and Pax7 expression in notexin-induced muscle injury in mice [16]. Similar upregulation is observed in primary satellite cells derived from mouse hindlimb muscle in vitro [16]. By contrast, treatment with 100 µg/kg Irisin fails to affect satellite cells within the vastus lateralis muscle in a hindlimb-suspended mice model [94]. This contradiction can be attributed to the dosage of Irisin or the injection pattern of it.

Irisin has been recognized as a pro-myogenic effector that promotes the differentiation and fusion of myogenic myoblasts through IL-6 signaling both in vitro and in vivo [16]. This pro-myogenic effect leads to significant hypertrophy in injured muscle with increased numbers of myofibers [16] and greater cross-sectional area (CSA) [16,94], as well as an enhancement of grip strength of uninjured muscle [16]. Irisin has also been shown to increase myotube number and fusion index in both C2C12 myoblast-induced and primary myotubes in vitro [16]. Moreover, Irisin promotes skeletal muscle hypertrophy by boosting protein synthesis and reducing protein breakdown [16,94]. Irisin also enhances mitochondrial density and size and promotes the transition of fast-type fibers towards the slow phenotype to counteract the reduction of slow fibers caused by unloaded-induced muscular atrophy [94]. Additionally, Irisin has been demonstrated to protect against fibrosis, myofiber necrosis, and sarcolemma instability in mice with dystrophic myofiber damage [19].

While Irisin has demonstrated potential in promoting muscle regeneration, further research is necessary to clarify conflicting findings on its effect on satellite cell activation and optimize therapeutic dosages with consideration of Irisin’s half-life. Intriguingly, combining Irisin with biomaterials capable of a sustained release may offer a promising approach for delivering Irisin to soft tissues like muscle.

#### 4.2.2. Osteogenesis

While Irisin has been found to activate the p38 and ERK signaling pathways, thereby promoting the proliferation and differentiation of osteoblasts in vitro, its role in regulating bone modeling and whether it can tip the balance towards bone formation under conditions such as osteoporosis or fracture remains a topic of debate.

Colaianni et al. were the first to demonstrate that Irisin has the potential to restore bone mass in osteoporotic mice induced by hind-limb suspension, which is consistent with a previous study showing that upon the same dose of 100 μg/kg of r-Irisin administered weekly for four weeks promotes healthy cortical bone formation [94]. The absence of mechanical loading was found to decrease the gene expression of osteoprotegerin (Opg) without affecting the expression of receptor activator of nuclear factor kappa-Β ligand (Rankl), resulting in an increased Rankl/Opg ratio, which usually a signal of osteoclast activation [179]. Interestingly, treatment with r-Irisin was shown to compromise the decrease in Opg expression without altering Rankl expression, hence leading to a similar Rankl/Opg ratio to that observed in mice under normal mechanical loading conditions [94]. This indicates a negative correlation between Irisin and osteoclastogenesis. A recent study showed Irisin can increase the number of osteoclasts within the callus during fracture healing in mice [180]. However, no evidence directly showed the osteoclast function was inhibited by Irisin with respect to bone remodeling/repair in vivo. On the contrary, in the OXV-induced osteoporotic mice, the ablation of FNDC5/Irisin can inhibit bone resorption as osteocytic osteolysis is eliminated by the inactivation of osteoclasts, suggesting a positive correlation between Irisin and osteoclastogenesis [20].

Administering 100 μg/kg of r-Irisin once a week for four weeks in a mouse model of disuse-induced osteoporosis resulted in decreased empty lacunae and prevented osteocyte apoptosis [129]. Irisin inhibited caspase activation in cortical bone and activated an ERK-dependent pathway including MAPK, ERK1 and ERK2, as well as upregulated the transcription factor 4 (Atf4) in osteocytes. B-cell lymphoma-2 (Bcl-2) proteins regulate apoptosis, and their relative expression with the Bcl-2 Antagonist X (Bax) protein determines cell survival. In vitro experiments on Mlo-y4 osteocyte-like cells showed that Irisin increased osteocyte viability and prevented caspase activation induced by dexamethasone and hydrogen peroxide via upregulation of the pro-survival Bcl2/Bax ratio [129].

Collectively, the regulation of osteoblasts, osteoclasts, and osteocytes by Irisin likely depends on the physiological state of the bone tissue, and the effects of Irisin may vary depending on the concentration or dosage administered. Further investigation is necessary to gain a comprehensive understanding of the therapeutic potential of Irisin for osteoporosis and bone healing by focusing on the bone multicellular unit.

#### 4.2.3. Neurogenesis

Neurogenesis, which primarily takes place in the subventricular zone and dentate gyrus within the hippocampus, plays a critical role in shaping the structural synaptic plasticity and neural network maintenance, thereby contributing to cognitive dysfunction recovery. Fndc5 expression level was increased during the early process of neurogenesis in mouse embryonic stem cells (mESCs) once treated with retinoic acid (RA) [181]. The RA-induced FNDC5 overexpression was further exclusively found in human neural tissues including the forebrain, hindbrain, myelencephalon, midbrain, and cervical spinal cord, suggesting the involvement of this gene in neural development [17]. Meanwhile, it also highlights the importance of FNDC5 in the development of the nervous system as FNDC5 knockdown significantly decreases the neural differentiation rate of mouse embryonic stem cells [182]. Regulation of FNDC5/Irisin in skeletal muscle was PGC-1α-dependent during exercise [9]. Similarly, neuronal Fndc5 gene expression is also regulated by PGC-1α and PGC-1α knock out mice show reduced FNDC5 expression in the brain [147]. FNDC5 has been identified as an important regulator of BDNF, which is a crucial neurotrophins upregulated by exercise and has an important role in neuronal cell proliferation, survival, and differentiation [183,184]. Forced expression of FNDC5 in primary cortical neurons elicits a concomitant enhancement in BDNF expression, whilst downregulation of FNDC5 via RNA interference confers a reduction in BDNF [147]. Uniformly, Choi and colleagues evinced the association between exercise-induced hippocampal neurogenesis in adult mice and heightened levels of both BDNF and FNDC5, resulting in ameliorated cognitive functionality in a murine model of AD [185]. Noteworthy, the PGC-1α/FNDC5/BDNF pathways can also be activated by flavonoid quercetin, resulting in neuronal adaptation in hyperbaric hypoxic rat hippocampus [186]. In addition to its direct expression and regulation in the cerebral cortex, Irisin generated in other tissues can also traverse the blood-brain barrier and stimulate the expression of BDNF in the hippocampus, suggesting that Irisin administered peripherally could induce some of the effects of endurance exercise on the neurogenesis [147]. However, it should be noted that hippocampal BDNF is not the sole factor that modulates neurogenesis in the hippocampus. One study suggests that Irisin, at a dose range between 50 to 100 nmol/L, affects hippocampal neurogenesis and induces mouse H19–7 hippocampal cell proliferation via activating neurogenesis-related signal transducer and activator of transcription 3 (STAT3) signaling [187].

Neuronal apoptosis, one of the main reasons for neurological deficits, can be counteracted by Irisin in multiple brain injury models. Increasing the levels of PGC1a, FNDC5, and BDNF has been shown to enhance neuronal cell survival and counteract the apoptotic effects on neurons [188]. Moreover, Irisin reduces apoptosis and increases BDNF protein, resulting in a significant reduction in infarct size and cerebral edema in animal models of stroke [27]. The anti-apoptosis function of Irisin is partially guaranteed by mitochondrial uncoupling protein 2 (UCP-2) protein during brain injury, resulting in elevated mitochondrial biogenesis in subarachnoid hemorrhage [29]. Furthermore, a recent study demonstrated the protective effect of Irisin on the peripheral nervous system (PNS) by ameliorating neuroinflammation-induced neuronal apoptosis in burn-related neuropathy, using recombinant adenovirus containing the Irisin sequence [189].

## 5. Conclusions and Further Remarks

Physical activity can trigger a broad range of adaptive mechano-sensing and -transducing responses, ultimately resulting in biogenesis for tissue regeneration and the suppression of degenerative processes. Although certain individuals may be unable to partake in exercise, they may yet obtain benefits from biomolecules that imitate its effects, such as Irisin. Indeed, Irisin has exhibited substantial potential as a remedial agent for degenerative disorders and displays numerous functions in the regulation of tissue regeneration. Notably, the mechanosensitive mechanisms that give rise to such an exercise mimic remain primarily obscure.

From a clinical standpoint, numerous contradictions and ambiguities remain to be addressed before classifying circulating Irisin as a prognostic or diagnostic biomarker for degenerative disorders. To begin with, the limited group sizes in these investigations alongside the possible impacts of confounding variables such as age, gender, and disease progression must be taken into account. In addition, the lack of standardized quantitative assays for Irisin precludes its identification as a biomarker, given the significant variability of reported reference values in serum, spanning from picograms to micrograms per milliliter.

With the utilization of state-of-the-art molecular technologies and disease models, significant progress has been made in the study of the therapeutic application of Irisin over the past decade. As clinical settings are replaced by laboratory investigations, Irisin has been found to play an essential role in the process of tissue regeneration, particularly in the case of age-related and injury-related deteriorating conditions. Beyond functioning as an anti-inflammatory and anti-apoptotic agent, Irisin coordinates the activities of various cell types involved in processes such as proliferation, differentiation, and apoptosis in damaged tissues including adipose, liver, cardiovascular, muscle, bone, and brain. However, considering the intricate nature of tissue regeneration, exploring the roles of Irisin in the complex process will require a multidisciplinary approach.

As we explore the secrets of the human body’s inner workings, it is essential to consider Irisin within the “exercise is medicine” framework, opening up new possibilities for public health. The ongoing study of the secretome and organokines contributes to an improved understanding of the mechanisms underlying the effects of exercise on overall health and disease prevention. As we uncover more about this fascinating area, we can continue to promote exercise as a viable solution for improving human health.

## Data Availability

The information that supports the findings of this study is available in this article.
